# The psychosocial impact of COVID-19 mitigation measures on frontline staff providing sexual health and family planning services in Kenya: a mixed-methods study

**DOI:** 10.1186/s12978-025-02089-9

**Published:** 2025-09-12

**Authors:** Ferdinand Okwaro, Abdu Mohiddin, Marleen Temmerman

**Affiliations:** Centre of Excellence in Women and Child Health, Aga Khan University, Nairobi, Kenya

**Keywords:** Psychosocial wellbeing, COVID-19, Healthcare workers, Sexual and reproductive health, Coping mechanisms

## Abstract

**Background:**

The recognition of the unintended impact of COVID-19 mitigation measures to the availability of sexual and reproductive health (SRH) services led to the initiation of mitigation and health systems support mechanisms within public health facilities by the Ministry of Health (MoH) in Kenya to maintain pre-Covid-19 levels of SRH service provision. These recovery mechanisms however concentrated on policy and infrastructural elements of service provision with limited attention given to the psychosocial impacts of COVID-19 on health care workers (HCWs). This paper examines the psychosocial impact of COVID-19 on front line family planning (FP) and sexually transmitted infections (STI) management HCWs and their coping mechanisms with a view to suggesting ways in which HCWs can be supported during future pandemics.

**Methodology:**

This paper employs a mixed methods approach with quantitative methodology analyzing data on the preparedness of the health sector to maintain service provision levels and qualitative data examining the mental and psychosocial states of HCWs who provide FP and STI health care services within Kenyan public health facilities.

**Results:**

Our main finding was that the psychosocial wellbeing of HCWs was majorly neglected in the government response mechanisms for the pandemic leading to burnout and depression, as well as HCWs absconding their duties in the initial stages of the pandemic, and avoidance of testing and disclosure of status as the pandemic progressed. Some of these mechanisms undermined the mitigation measures by the government and put patients and colleagues at risk of infection by HCWs whose COVID-19 status remained unknown.

**Conclusions:**

We recommend that future responses include mechanisms that address the psychosocial wellbeing of HCWs as a core element of the response for effective management of pandemics. In the case of new and unprecedented pandemics such as COVID-19, it is important that HCWs are provided with accurate and timely information about the pandemic as well as to what is expected of their conduct in service delivery and in the promotion of a culture of risk reduction.

## Abstract (plain language summary)

This study employed quantitative and qualitative research methodologies to examine the effect of COVID-19 on the healthcare workers (HCWs) psychosocial health and their coping mechanisms. HCWs are critical in the management of epidemics since they are involved in outbreak control and the treatment of patients. Maintaining their physical and psychosocial health is crucial for the success of the infection prevention and control (IPC) mechanisms during an epidemic. The COVID-19 outbreak led to the initiation of measures such as the use of IPC materials, social distancing and delimitation of patient numbers by HCWs. There was however little attention given to the psychosocial well-being of health care workers with very few measures adopted to support healthcare workers mental health. The increasing cases of COVID-19, heavy workloads, shortages of personnel protective equipment, information overload and contradictions and a lack of public support from a suspicious populace caused immense mental stress to HCWs. In the absence of a collective government mental health intervention, HCWs adopted coping mechanisms such as refusing to test for COVID-19 and hiding their COVID-19 status to avoid stigma and ostracization by the community. These and other defense mechanisms put both patients and colleagues at risk of infection. This study recommends that during pandemics special attention be given to the psychosocial health of HCWs through provision of counselling services and timely and accurate information alongside other support measures if the epidemic control mechanisms are to be successful.

## Introduction

The nature of the work and work environment have for long been recognized as important contributors to employees mental and physical wellbeing and it has become common practice for organizations to implement psychological interventions to promote the job performance and wellbeing of their employees [1]. Psychosocial wellbeing is however not impacted on or confined to the work environment alone: the work/home interface is recognized as an important source of stress since the demands of the home environment are significantly related to the occupational stress [2, 3]. Consequently, researchers include the work/home interface among the six main factors that are common sources of stress for all jobs, the other factors being, factors intrinsic to the job, career development, role-based stress, relationships at work and organizational structure and climate [2].

Although not so much research has been done to compare stress levels of different occupations [2], professions such as the health care professions, that involve human contact and rapid decision making have been described as most stressful [4, 5]. Moreover, pandemics such as the Ebola and COVID-19 have been identified as occurrences that exacerbate an already dire situation especially in low-income settings grappling with scarcity of human and capital resources [6–8]. The mental burden among HCWs, in times of pandemics is often attributed to the rapid increase of infected cases, deaths, absence of effective medications or vaccines, extensive media coverage, massive workload resulting in long hours of work, lack of personal protective equipment (PPEs) leading to anxiety about the risk of infection, feelings of inadequate support and exposure to fake news and rumors in an environment of rapidly changing knowledge about the nature of the pandemic [9, 10]. Previous research conducted on other infectious diseases such as the severe acute respiratory syndrome (SARS), the Middle East Respiratory Syndrome (MERS) and the Ebola virus showed that HCWs suffered significant stress both during and after the pandemics [8, 11].

This paper examines the psychosocial impact of COVID-19 and its mitigation measures on front line FP and STIs HCWs in Kenya. It uses quantitative and qualitative data collected in the course of a health systems analysis project that evaluated the barriers to availability, utilisation and readiness of sexual and reproductive health services in COVID-19 affected areas in Kenya. The study was conducted across 12 health facilities in Kenya, evenly distributed in the counties of Nairobi, Mombasa and Kilifi.

### The COVID-19 pandemic

The first case of COVID-19 in Kenya was reported on the 13/3/2020 [12]. While at the beginning cases remained low, they continued to increase steadily reaching a first peak in July 2020 with approximately over 800 cases recorded daily. The Ministry of Health (MoH) provided daily updates of the COVID-19 situation, detailing among other statistics, the number of new cases reported, total number of tests conducted, distribution of cases within geographical locations (counties) and number of recoveries [12].

On the basis of the first case reported and events from other parts of the world, the MoH in Kenya announced a raft of nationwide precautionary measures to curb the spread of the disease. These included, information, education and communication, hand hygiene, respiratory etiquette, masks for everyone including the HCWs, isolation and treatment of sick individuals, monitoring symptoms of healthy contacts, traveller health advice, environmental fumigation and cleaning, social distancing and avoidance of crowds, institutions’ closures, restrictions on the use of public transport, workplace closures and public health quarantine for asymptomatic individuals and/or isolation for ill individuals [13]. In particular, the health sector was supported with training, PPEs and isolation and treatment centres in referral facilities [13].

As the different measures were implemented, HCWs in Kenya and globally remained at the forefront fighting the progression of the virus through treating of COVID-19 cases, in addition to other pathologies that occur in the healthcare centres on a daily basis [14, 15]. While uninfected members of the population were asked to stay at home as one of the mitigation measures, HCWs were required to go out to fight the progression of the disease, thereby exposing them to high levels of stress and work overload affecting their physical, mental and social health. Since COVID-19 was new to humans, the uncertainty of the progression of the disease, the rapidly changing information disseminated by the World Health Organisation (WHO) and other authoritative scientific institutions such as the Centres for Disease Prevention and Control (CDC), misinformation on social networks, and different levels of severity of disease placed many HCWs in a situation of adversity [16, 17].

Several studies have been conducted to explore the manifestations of psychological stressors among HCWs across the globe, [18–25]. In Kenya, a number of studies [26–28] have equally examined the prevalence of mental disorders among HCWs. However, many of these studies with the exception of Shah et al. [18] have employed quantitative methodologies to measure the prevalence of mental health symptoms among HCWs employing standardized questionnaires with preset parameters of mental health determinants. By contrast, this study employed a mixed methods approach, using in-depth interviews that allowed the HCWs to, in their own words, express the stressors engendered by the pandemic in the continuum between their homes and workplace.

### Methodology

This study was carried out on two levels: at the individual level, where the perspectives of clients and HCWs were examined using qualitative methods of data collection (in-depth interviews) and at the health facility level involving a quantitative assessment of infrastructure availability and readiness to provide SRH services using the service availability and readiness assessment (SARA) tool. The results presented here will be based mainly from the IDIs with healthcare providers, augmented by quantitative data.

### Study settings and facilities levels

This study was conducted in three counties in Kenya, namely Nairobi, Mombasa and Kilifi. These three counties were among the first ones to record COVID-19 cases and were subsequently placed under lockdown, with transport in and out of them restricted. In addition, while movement within the three counties was allowed, specific localities within Nairobi and Mombasa, i.e. Eastleigh in Nairobi and Old town in Mombasa, were placed under total lockdown at the beginning of the pandemic.

Twelve health facilities were selected as sites for these study i.e. 4 in each county and distributed to cover the urban, peri-urban, rural and youth friendly service facilities. Health service provision in Kenya is organized along six tiers: community (level 1), primary care dispensaries (level 2) health centres (level 3), subcounty hospitals (level 4) county hospitals (level 5), and tertiary referral hospitals (level 6) [29]. The four facilities selected in each county were distributed between levels 2 and 5.

### Study participants selection

HCWs were purposively selected to participate in the IDIs and in the health facility assessment. The interviews were conducted during the pandemic and after the pandemic in order to assess whether there were any differences in impact at these two times. Although 2 HCWs per health facility were selected to participate in the IDIs, only 22 of these were available during the pandemic and 18 after the pandemic. The research team however determined that saturation point had been achieved with these sample sizes. Out of the 40, 10 HCWs provided STI care while 30 provided FP services. The selection criteria included only those 1) who delivered SRH services, 2) were most knowledgeable about readiness and availability of SRH services 3) were stationed in the SRH clinic and had been working at the clinic for at least 6 months prior to the pandemic. Participation was voluntary and only the HCWs who accepted to participate by signing an informed consent form were interviewed.

HCWs were interviewed using an IDI guide that covered WHO’s six building blocks framework with a focus on the delivery of contraception, comprehensive abortion care, STI prevention and treatment and gender-based violence care and support services during COVID-19; and their perceptions on the roles and responsibilities of different parties for providing these services in the context of COVID-19; health system capacity to provide good quality of care for people during COVID-19; training needs, attitudes, biases about contraception, abortion, STI and GBV in the context of COVID-19; and perceived psychosocial effects on men and women, their families and local communities. In describing their psychosocial health, the narrative approach was employed in which the HCWs described their states in their own words as opposed to using parameters commonly defined in mental health literature [30]. The IDIs were conducted by three qualified researchers with experience in conducting IDIs. The IDIs were conducted at two times; during the outbreak between August and December 2021 and after the outbreak between April and June 2022). All our informants were female as they were the ones who mainly provided FP and STI services in the facilities visited and were available for interview. All the IDIs were audio-recorded and transcribed. The audio recordings were destroyed after verification by the first author.

All the facilities selected in this study offered FP and STI management services by different categories of HCWs. Level 2 facilities had a nurse/midwife, public health technicians and community health extension workers (CHEW) while level 3 had a doctor or clinical officer over and above the HCWs available at level 2. HCWs at higher level facilities ( 4 and 5) included doctors (including specialists), clinical officers, public health officers and nurses/midwives. All FP HCWs were nurses/midwives while 4 and 6 of the STI management HCWs were doctors and clinical officers respectively.

### Data analysis

An inductive approach was used for the analysis to allow for the emergence of the themes and codes from the data. The analysis of the qualitative data was based on the verbatim transcripts following the general approach of content analysis according to the steps suggested by Elo & Kyngäs [28]. The analysis involved reading through the verbatim transcriptions of the interviews several times to gain an understanding of what was being expressed by the participants, noting down emerging themes and patterns in the data and condensation of the data. The researchers decided on the unit of analysis and creation of units of meaning, which were condensed into codes, while ensuring the core meaning was not lost or distorted. The codes were then used to generate categories and condensation of related categories into themes that convey the meaning of the data.

Analysis of the quantitative data used the presence of the tracer items for the provision of these services, such as availability of guidelines, staff and availability of essential commodities. Quantitative data from the facility assessment survey was entered into an electronic database using a statistical analysis software/package. Descriptive analysis was used to illustrate the basic characteristics of the different facilities, including the monthly number of clients, types of procedures provided, number of medical staff and stocks of drugs.

## Results

### Health systems readiness to provide FP and STI services

The SARA tool was administered at facility level and responded to by the most knowledgeable HCW to measure the availability and readiness of the health care facilities to provide services during the COVID-19 pandemic using a number of indicators as can be seen in Table 1 below. All HCWs stated that the country had defined a national SRH essential health services package prior to the onset of COVID-19. When asked about the status during the pandemic, 75% indicated that the country had identified a core set of essential health services to be maintained during the COVID-19 pandemic. Majority of the respondents (66.7%) stated that the government did not allocate additional funding for assuring the maintenance of essential health services. 75% of the HCWs had received the latest WHO and other technical guidance documents, and all had shared the guidelines with other health care service providers.

**Table 1:** COVID-19 SRH service provision guidelines and support to health facilities

Policy guideline	Availability *n* = 12		
	**Yes**	**No**	**Don’t Know**
Is there a National SRH package prior to COVID-19	100%	-	-
Is there a core set of essential health services to be maintained during the COVID-19 pandemic	75%	25%	-
Is there additional government funding for COVID-19	66.7%	33.3%	-
Have you received latest WHO COVID-19 guidelines	75%	25%	-
Are the guidelines being used to guide service provision?	100%	-	-

HCWs were also asked about their perception of the government policy regarding selected healthcare services. Most of the FP and STI patients would be seen at the outpatient clinic and as the Table 2 below shows, 75% of the HCWs thought that the government expected them to continue to provide outpatient service, 16.7% were unsure and 8.3% thought that all outpatient services were suspended.

**Table 2:** HCW’s perception on government policy regarding selected health care services (*N* = 12)

Type of service	Level of maintenance *N* = 12			
	**Normal**	**Limited access**	**suspended**	**Don’t know**
Outpatient care	75.%		8.3%	16.7%
Inpatient care	33.3%	16.7%		50.0%
Emergency unit services	83.3%	16.7%	0.0%	0.0%
Pre-hospital emergency care services	75.%	8.3%		16.7%
Community based care	58.3%	25.%	8.3%	8.3%
Mobile clinics	8.3%	16.7%	8.3%	66.7%

### Disruption to FP services and availability of commodities

58.3% of the HCWs stated that FP service provision was partially disrupted while 41.7% stated that the services at their facilities were not disrupted at all. The table 3 below shows the availability of FP commodities at the facilities surveyed.

Six contraceptive commodities were stocked in all the facilities sampled at the time of conducting this survey: these are the combined oestrogen progesterone oral contraceptive pill, male condom, female condom, implant (subdermal implant), Copper IUD and the Levonorgestrel Intrauterine Device. On the other hand, the vaginal ring was not stocked in any of the facilities sampled while the emergency contraceptive pill was stocked in only 33% of the facilities sampled.

### Availability of STI services

All facilities included in the study offered diagnosis and treatment of STIs services. Additionally, all facilities had the national guidelines for the diagnosis and treatment of STIs available in the facility on the day of the survey. With regard to prescribing treatment for STIs, 91.7% [12] of the health care facilities diagnosed and treated STIs (Table 3).

**Table 3:** Availability of contraceptives at the facility (*N* = 12)

Commodity	Availability *N* = 12	
	**Yes**	**No**
Combined oestrogen progesterone oral contraceptive pill	100%	0.0%
Progestin-only contraceptive pill	66.7%	33.3%
Combined oestrogen progesterone injectable contraceptive	8.3%	91.7%
Progestin only injectable contraceptive (DMPA or NET-EN	75.%	25.%
Male condom	100%	0.0%
Female condom	100%	0.0%
Emergency contraceptive pill	33.3%	66.7%
Cycle beads for standard days method	83.3%	16.7%
Vaginal ring	0.0%	100.0%
Implant (subdermal implant)	100.0%	0.0%
Copper IUD	100%	0.0%
Levonorgestrel Intrauterine Device	100%	0.0%
Other natural method	58.3%	41.7%
Diaphragm	16.7%	83.3%

HCWs were asked about the kind of STI tests offered at their facilities. The table below shows the number of health facilities in this study providing selected STI services.

Table 4 above below shows that the following services were available in all the facilities: syphilis rapid test, HIV rapid test, urine protein dipstick testing, urine glucose dipstick testing and urine ketone dipstick testing. Syphilis dark field Microscopy was lacking in 91.7% of the facilities while 33.3% of the facilities did not have the dry blood spot (DPS) collection for HIV Viral load EID service.

**Table 4:** Availability of STI/HIV testing (*N* = 12)

Type of test	Availability at facility	
	**Yes**	**No**
Syphilis rapid test	100%	0.0%
HIV rapid test	100%	0.0%
Syphilis dark field Microscopy	8.3%	91.7%
Urine glucose dipstick testing	100%	0.0%
Dry Blood Spot (DPS) collection for HIV Viral load EID	66.7%	33.3%

### Psychosocial distress in the provision of FP and STI services

Most HCWs stressed the fact that with the exception of oral contraceptives provision, most FP and STI care brought them into close proximity to patients and therefore to possibilities for contagion in the absence of appropriate PPEs. Perceptions of vulnerability to psychosocial distress were elicited using an IDI guide with a section specifically asking HCWs to assess their psychosocial health during the COVID-19 pandemic. The themes discussed here covered the expressions of psychosocial distress, sources of stress, nature of the stressor and coping mechanisms employed by HCWs. The stressors identified by the HCWs included the health care profession as a stressor, lack of information and fear of contagion, struggles balancing the work and home interface, absence of government support and discrimination and ostracization by the community.

### Expressions of psychosocial distress

HCWs used a number of expressions to describe their psychosocial health during the COVID-19 pandemic. These included, depression, stigma, trauma, extreme fear, anxiety, being overwhelmed, emotional and physical trauma, loneliness, chaos, ostracization by community, torment and burnout. The following excerpts illustrate some of the statements used by the HCWs.

*‘I felt constrained, like my freedom was curtailed’* (FP – HCW: 006).

*‘I was tormented, I was tormented, the only thing I kept anchoring on was to let God be my protector’* (STI – HCW: 015).

*‘I could not visit friends, because you fear if you go there and someone becomes sick and then you are told you are the one who came there with COVID-19, so it made us very lonely’* (FP – HCW: 013).

*‘Emotionally it was a bit of trauma, physically it was also overwhelming, sometimes we worked from 8 am to 8 pm.. it was burn-out, it was emotional trauma’* (FP- HCW: 016).

Most HCWs were however quick to point out that the psychosocial distress declined when more information about COVID-19 was available, guidelines from the ministry were provided and there was a consistent supply of the right type of PPEs.

### Sources of psychosocial distress

#### The healthcare profession

HCWs mentioned the increased risk of infection inherent in direct exposure to patients with an infectious pathology as one of the biggest stressors and risk to their physical and psychological wellbeing. Especially at the beginning of the pandemic when there were no PPEs and knowledge of the mode of spread of COVID-19 was unclear, many HCWs lived in fear of contagion and of infecting their own family members. The comment below by a HCW exemplifies this situation:

*You know our profession, especially the fact that in some cases you must observe patients at very close range. COVID-19, being new disease, it really affected health workers psychologically because …. Eh .. people could fear… could fear attending to the patients, people could hear that a client is coming, perhaps suspected to have COVID-19, some health workers used to run away, but at the moment things have changed* (FP-HCW: 002)

#### Lack of guidelines and fear of contagion

In the initial months of the pandemic, HCWs observed that their biggest source of stress and anxiety was the lack of information and guidelines from the Ministry of Health about COVID-19 and how they were expected to provide services while protecting themselves. These two comments below exemplify this.

*For us the biggest contradiction was that while everybody else was instructed about what to do, nobody told us anything. We were left to figure out for ourselves what to do. Either go to work or fail to go to work and be fired. It was so stressful to have to choose between your job and your life.* (STI-HCW:034)

*It was overwhelming, because I remember when one of our staff got positive.. when they went for isolation, we were very afraid.. there were no guidelines about what we should do, because now we are working here and we are going back home, so it was a bit of a trauma, and at that time I was breastfeeding, so it was a bit hectic emotionally.* (FP-HCW:007)*.*

#### The work and home interface

The expectation for female HCWs to juggle between work and home responsibilities was yet another source of anxiety for the HCWs. The problem was exacerbated when family members were anxious and occasionally hostile to the HCWs as the quote below shows.

*I had a family back at home and I would come to work and leave the work place and go back home. There was nowhere designated where you could be housed for this period away from your family. I had a personal experience in that it actually impacted my family life when my husband said, ‘No, you are going to give us an infection. That’s why I keep talking of the psychological care. It was not there* (STI-HCW: 015).

*Because I am working at the hospital, one of my sons said, ‘Mum you are going to get COVID and you are going to infect us’. Of course I would laugh it off and reassure him but at the back of my mind, you know …. I knew some people will see you as the spreader because you are working in the hospital* (FP-HCW:014)

Some of the HCWs who could afford extra space in their households or could afford rented apartments isolated themselves to avoid infecting other family members especially those with comorbidities as the quote below shows.

*I stay with my mom and she is diabetic and so I had to make sure she stays on one side [of the house] and fortunately for me we had two toilets and bathrooms, so I would use mine specifically and when I come home, you know, they would throw me a leso (wrap round cloth) through the window and I had to remove any clothes I had from the hospital, wash myself, I used to wash my unforms outside the house* (FP-HCW:012).

### The social undesirability of the HCW

Discrimination and ostracization in the community were yet other sources of stress for HCWs. Some HCWs complained that members of the community shunned them at public functions for fear they would infect them on account of their daily contact with patients at the hospital who might be infected with COVID-19.

*There was the stigma from people, now there was a perception.. ‘Oh.. this one is a nurse, she is a health care worker, you know she will bring us Corona from work. People looked at health care workers as sources of COVID. I think it is terrible, personally I think it is terrible, people were getting depressed, people got lonely* (FP-HCW:014).

### HCWs as agents of cultural disrespect

Similar to the social undesirability, HCWs complained that enforcing the COVID-19 infection prevention and control (IPC) measures such as restricting number of patients to the facility, and at funerals was interpreted by community members as disrespectful to cultural traditions. This led to further ostracization by members of the community.

*There was this person who died from COVID and you know that time they were being buried in plastic bags, so the community felt at that time that.. you know there is that honor for the dead, the community felt that they had thrown away their person because they were buried by public health officers.. that was very stressful for the community here and they took their stresses on us.* (STI-HCW: 006).

#### Lack of government support

Most HCWs complained that there was no government support for their psychosocial wellbeing during the COVID-19 period. Instead HCWs were left to their own devices leading to feelings of rejection and depression. Female HCWs were asked whether they received any extra supplies of PPEs or sanitary towels in view of their double roles as HCWs and home responsibilities as mothers and wives. All HCWs interviewed stated that they did not receive any extra supplies. Performing these double roles and the extra care required of them was a cause of stress, anxiety and burn out as the quote below show.

*The government in Kenya failed health care workers in terms of psychosocial care and self-care for workers where after working in a COVID centre, or after working in a hospital, they need to undergo what we call debriefing sessions which were not provided* (STI-HCW: 002)

### Coping mechanisms

#### Avoidance of testing and disclosure of COVID-19 symptoms

Faced with the strict and often deplorable quarantine conditions, inability to isolate due to resource constraints, (lack of extra rooms in their homes to isolate) stigmatization and ostracization by the community, many HCWs reported that they concealed any symptoms that would lead to the suspicion that they had COVID-19 (high temperature, coughs, shortness of breath, fatigue or headaches) and never went for testing. Many of the HCWs who declined to go for testing stated that they believed they had contracted COVID-19 severally during the pandemic but actively concealed their suspicion to their supervisors and their colleagues for fear that they would be asked to take a COVID-19 test, a positive result of which would lead to them being quarantined in public facilities with deplorable conditions.

*When COVID was COVID, we used to get even a flu, maybe you go test, you find you are positive, but due to stigma, you will not tell anyone. People really hid themselves. You couldn’t say you had COVID because if you did, contact tracing [would] start, your neighbours would start running away from you.... so you decide, let me just take medications and….stay at home for a few days and resume work* (STI-HCW:010),

Many HCWs stated that the practice of concealing one’s suspicions of COVID-19 infection was common in their facilities as the quote below shows.

*I know quite a number though they were never hospitalized. They just took drugs. But most staff, we had very bad flu.. those symptoms, no sense of taste, Yeaah, quite a number of people fell ill but did not test* (STI-HCW:010).

#### Reliance on faith

Most HCWs interviewed observed that there were no formal mechanisms set by the MoH for counselling and many of them depended on their strong will and reliance on faith for consolation.

*I was tormented, it is only that the only thing I kept on anchoring on was God, let him be my saviour, because it reached a point where it was beyond us. Ours was just that as long as you are on duty, but how you go nobody knows or cares. ..imagine at a point where you are on duty, you don’t have protective gear, some of them you have run out of, and the only thing you can afford is a mask, and if you stay at home and don’t come to work, you have to explain why you are not on duty, then you go back home and your family is telling you, you are bringing them an infection…it was chaotic, it wasn’t a good scene* (FP-HCW: 015).

## Discussion

This study employed a narrative approach to the examination of the psychosocial health of HCWs in view of the critique of psychometric tests as narrow and unsuitable for societal level examination of well-being [30, 31]. These critiques instead, advocate for a conceptualization of psychosocial wellbeing through a subject who can freely speak their mind instead of one that is silenced and ‘replaced by force choice methods regarding both items and response formats’ [30]. Our study corroborates the findings from other studies which found out that a combination of the absence of PPEs, inadequate training of HCWs (that excluded training on psychosocial wellbeing) and a lack of clear guidelines and resources all had a negative effect on the psychosocial wellbeing of the HCWs [32, 33]. The absence of clear guidelines was however not a Kenyan issue alone since, equally, authoritative global sources of authentic information, such as the WHO and the CDC were issuing new information and guidelines almost on a daily basis [17]. This conflicting information, images from the West where the pandemic was already causing massive deaths and morbidity, were readily accessible to HCWs in Kenya through global information communication channels, which led to stress and anxiety among HCWs.

This study corroborates the findings from other studies [2] that the work/home interface was one of the major sources of stress for all jobs, especially for health care professionals during highly infectious pandemics. While some studies separate the work and home environments as distinct domains of stress production [6, 24, 34], this study conceives the work/home interface as a continuum of interacting factors that produce stress to HCWs. The work environment that many HCWs operated in was characterized by extreme anxiety and fear of infection related to the lack of PPEs and guidelines on service provision. The prospect for social support from the home was nullified by the requirement for social distancing, stigma and ostracization by family and other household members. All HCWs spoke of the contradiction between the desire to protect their family members by staying away from them, especially their children from infection, and the need for the same family for emotional support to relive stresses precipitated by the work environment. Exclusion and avoidance of contact with wider community members by HCWs during epidemics was recommended highly to stem the spread of the virus [35]. The absence of government or employer led stress and anxiety relief mechanisms coupled by the impossibility of getting the same from their social networks (family and community) caused HCWs to feel that that the recognition of their work had been neglected thereby leading to dissatisfaction with their profession [36].

HCWs are known to employ several coping mechanisms during pandemics. These mechanisms can be healthy, such as healthy eating, exercising, religious activities and spending time with pets and loved ones [18]. However HCWs are also known to adopt unhealthy coping mechanisms such as consumption of unhealthy foods and increased substance usage (e.g. alcohol and marijuana (ibid). In this study, HCWs reported refusal to test and disclosure of their COVID-19 status as a coping strategy to deal with stigma, ostracization and being subjected to harsh quarantine conditions. Lack of resources in their homes for isolation were also highlighted as the reasons for their reluctance to test and/or disclose their COVID-19 status. The refusal by HCWs to test for COVID-19 even when they showed signs of what could be COVID-19 or had come into contact with a confirmed COVID-19 went against the basic infection prevention measures stipulated during the pandemic and thus endangering patients and other HCWs. HCWs should be encouraged and motivated to ensure that they are fit for duty and free of any communicable diseases. The health care service should devise mechanisms that ensure that HCWs comply with the stipulated prevention infection control measures. It is important therefore for future pandemics that the totality of HCWs providers experience with the changed circumstances of both their work and home environment be taken into account while designing interventions and health workers be supported to ensure that they do not adopt counterproductive coping mechanisms that undermine the quest to stem the spread of the pandemic.

This study paid special attention to female health care providers who are often on the frontline of the health system response and who comprise 70% and up to 90% of the health and social care workforce respectively [37, 38]. Studies have pointed out that nurses, who are often predominantly women, face greater family burdens which add work stress to their daily life stress [36]. HCWs in this study who were all female reported that they did not receive any extra supplies or support from their employers. Intervention and mitigation measures need to evaluate in-depth the gender variables in the stress and anxiety experienced by female HCWs since healthcare professions tend to have a greater female disposition [37, 39].

The psychological wellbeing of HCWs have far reaching effects on the quality of care that patients receive. HCWs stress at work and burnout have been found to lead to an increase in medical errors [40], [51] Increased stress and burnout have also been associated with hospital acquired infections [41] and lower levels of patient satisfaction [42]. In addition to the stresses and burnout associated with health care work in general, studies on the impact of the COVID-19 pandemic and its mitigation measures showed that the pandemic had an unprecedented impact on the Psychosocial health of HCWs on the front lines of pandemic response efforts [18, 26, 28, 34, 43]. And while the impact of the pandemic on frontline healthcare workers may have been mitigated through responses from occupational health and safety units in high income countries, [43] response to the pandemic and to the protection of health care providers physical and mental health in lower income countries (LICs) like Kenya was haphazard especially in the early months of the pandemic. Feierman [44] has described African health care systems as living in a state of perpetual normal emergency due to a lack of investment and dependence on support from multilateral and bilateral donors, a phenomenon termed as the experimentation of health care provisioning in Africa [45]. Pandemics such as the COVID-19 and the Ebola exposed this sorry state of affairs of many African health systems who ended up depending on meagre national resources augmented by uncoordinated donations from the international community who gave the response to COVID-19 a semblance of resilience.

The COVID-19 pandemic exposed this precarious state of Kenya and other African health systems in which despite the earlier warnings regarding the rapid global spread of the SARs-CoV-2, the discovery of the virus in Kenya was characterized by a lack of preparedness exemplified by the lack of PPEs in the earlier days of the pandemic. This led some HCWs to flee from health care facilities any time a COVID-19 suspected case was detected. The lack of PPEs especially in the early stages of the pandemic and the consequent heightened fear of contagion and transmission to family members have been identified in this study and several other studies as sources of stressors for HCWs. Unlike in the HICs like the USA where Cai et al. [17] identified the rapidly changing PPEs donning and dolling protocols as a cause of stress, HCWs in Kenya did not have access to PPEs which caused anxiety and fear of infection.

### Strengths and limitations of the study

The strength of this study is the employment of a mixed methodology combining qualitative and quantitative data collection techniques. These enabled the collection and triangulation of multiple data that provide a comprehensive view of service availability and disruptions during the COVID-19 pandemic. Data was collected through in-depth interviews with HCWs and clients as well as from health records form the facility. The limitation is the fact that this study compares pandemic and post-pandemic situation and not the pre-pandemic evaluation. A comparison between the pandemic and pre-pandemic situation would provide a better picture since the post-pandemic might as well be affected by the very effects of the pandemic that the study measures.

## Conclusion

The COVID-19 pandemic had a high negative impact on the psychosocial wellbeing of HCWs in Kenya. While several studies have evaluated the impact COVID-19 employing standard psychometric indicators, this study employed a narrative approach that allowed the HCWs express their experiences without the limitations of predetermined psychometric categories. By so doing the HCWs identified the predominant sources and manifestations of stress and anxiety in an intricate web of relations in their work and home domains in a context of resource deprivation. Extreme fear and anxiety in a resource poor environment where the government is unable to provide psychosocial support, and where HCWs are incapable of enacting infection prevention measures such as isolation may lead to the adoption of counterproductive coping mechanisms such as the refusal to test and declaration of suspicious symptoms. The context within which interventions measures are enacted are thus as important as the intervention measures themselves if the efforts at controlling the spread of an epidemic are to achieve their intentions. This study recommends that future pandemic response factor in context specific mechanisms that address the psychosocial wellbeing of HCWs as a core element of the response for effective management of pandemics.
